# Schiff Base Functionalized Cellulose: Towards Strong Support-Cobalt Nanoparticles Interactions for High Catalytic Performances

**DOI:** 10.3390/molecules29081734

**Published:** 2024-04-11

**Authors:** Hicham Aitbella, Larbi Belachemi, Nicolas Merle, Philippe Zinck, Hamid Kaddami

**Affiliations:** 1 IMED-Lab, Team of Organometallic and Macromolecular Chemistry-Composite Materials, Department of Chemical Sciences, Faculty of Science and Technology, Cadi Ayyad University, Marrakech 40000, Morocco; 2 Unité de Catalyse et Chimie du Solide, UMR 8181, University Lille, CNRS, Centrale Lille, University Artois, F-59650 Villeneuve d’Ascq, France; 3 Sustainable Materials Research Center (SusMat-RC), Mohammed VI Polytechnic University (UM6P), Lot 660, Hay Moulay Rachid, Ben Guerir 43150, Morocco

**Keywords:** cellulose oxidation, Schiff base, supported catalyst, salen, reduction, cinnamaldehyde, selective hydrogenation

## Abstract

A new hybrid catalyst consisting of cobalt nanoparticles immobilized onto cellulose was developed. The cellulosic matrix is derived from date palm biomass waste, which was oxidized by sodium periodate to yield dialdehyde and was further derivatized by grafting orthoaminophenol as a metal ion complexing agent. The new hybrid catalyst was characterized by FT-IR, solid-state NMR, XRD, SEM, TEM, ICP, and XPS. The catalytic potential of the nanocatalyst was then evaluated in the catalytic hydrogenation of 4-nitrophenol to 4-aminophenol under mild experimental conditions in aqueous medium in the presence of NaBH_4_ at room temperature. The reaction achieved complete conversion within a short period of 7 min. The rate constant was calculated to be K = 8.7 × 10^−3^ s^−1^. The catalyst was recycled for eight cycles. Furthermore, we explored the application of the same catalyst for the hydrogenation of cinnamaldehyde using dihydrogen under different reaction conditions. The results obtained were highly promising, exhibiting both high conversion and excellent selectivity in cinnamyl alcohol.

## 1. Introduction

Metal nanoparticles (NPs) have become very popular due to their exceptional properties and essential uses in a variety of applications. They are recognized as efficient catalysts, both in academic research and in industry. A wide variety of late transition metal NPs have been widely used for a number of organic reactions. Earth-abundant cobalt, especially in the form of nanoparticles, has demonstrated remarkable effectiveness as a catalyst. It stands out due to its lower cost compared to other noble metal catalysts like Pd, Pt, and Au [1]. For example, CoNPs have shown promising activities in Fischer–Tropsch synthesis [2,3], alcohol, and carbon monoxide oxidation [4,5,6]. However, one of the major challenges encountered in the preparation of CoNPs is the issue of aggregation, which can significantly compromise the catalytic performance of nanoparticles. Aggregation refers to the undesirable agglomeration of CoNPs, leading to a reduction in surface area and a decrease in catalytic activity [7]. This phenomenon hampers the efficient utilization of CoNPs and poses a major obstacle to achieving optimal catalytic performances. This phenomenon can be solved by selecting ideal support materials that exhibit a large surface area, excellent stability, high porosity with good accessibility, and the presence of versatile functional groups for strong interaction with the metal. Therefore, the development of effective supports for the immobilization of CoNPs is highly desirable. Consequently, various supports such as mesoporous silica [8,9], graphene, carbon nanotubes [10,11], zeolites [12,13], and polymers [14], have been employed for the immobilization and stabilization of metal NPs. These supports further facilitate separation and open the way to catalyst recycling or continuous process in order to reduce production costs and minimize waste generation, particularly in industrial applications.

Cellulose is an attractive support for catalytic systems thanks to its high surface area, large number of potentially functionalizable hydroxyl groups, and biodegradability among other properties. Regarding native cellulose as support for metal particle catalysts, the relatively weak interactions between the surface and the particles cannot totally prevent catalyst deactivation by sintering and it is why research efforts have focused on cellulose modification. Fortunately, there are a plethora of modification methods to improve its performance for specific applications, such as crosslinking [15,16], grafting of polymer [17,18,19,20], enzymatic modification [21], chemical and physical modification [22,23], and TEMPO oxidation [24]. The process of periodate oxidation that transforms the 2,3-dihydroxyl groups into 2,3-dialdehyde groups through a selective cleavage of the C2 and C3 vicinal hydroxyl groups [25,26] is an example of such modifications. The resultant dialdehyde cellulose holds significant potential to be used in many applications after modification or blending with other materials [27]. For example, it allows the introduction of different substituent groups on its backbone such as primary alcohols [28,29], carboxylic acids [30,31,32], cationic groups [33,34,35,36] organometallic species, imidazolium salts [37,38], and imines [39,40]. Regarding these later modifications, dialdehyde cellulose (DAC) can react with amines via a condensation reaction, leading to Schiff bases and introducing imine functionalities. Due to their particular structure, the cellulose-based Schiff bases are valuable for recovering transition metals, and cationic and anionic dyes [41,42,43,44].

In this study, the immobilization of the cobalt nanoparticles on the surface of a Schiff-based functionalized cellulose is reported. The latter is obtained by oxidation of cellulose leading to dialdehyde and further condensation with orthoaminophenol. In order to assess the stability of our catalytic system we turn our attention to two appealing reactions that allow us to challenge our system due to the reaction conditions. The first one was performed in aqueous media, which is known to favor aggregation [45,46,47]. The second is performed in an organic solvent but under hydrogen pressure and higher temperature, which is also known to favor sintering [48]. Thus, our supported catalyst was first evaluated for the reduction of 4-nitrophenol to 4-aminophenol based on the fact that CoNPs have been shown to be effective in nitroarene reduction catalysts [49,50]. Nitrophenols are the most refractory pollutants that can be found in chemical industry wastewater [51], resulting particularly from pesticide, herbicide, and insecticide manufacturing and degradation processes [52]. Additionally, the synthesis of dyes and the pharmaceutical industry also account for their production [53]. These toxic compounds cause skin irritation and allergies [54]. Secondly, the selective catalytic hydrogenation of the cinnamaldehyde to cinnamyl alcohol under H_2_ was also studied. Selective hydrogenation of cinnamaldehyde (CAL) serves as an important and representative model reaction, as both the carbonyl group (C=O) and the carbon–carbon double bond (C=C) of the molecule can be involved in the hydrogenation. However, from a thermodynamic perspective, the hydrogenation of the C=C group of the enone is more favorable than that of the C=O because C=C bond energy (615 kJ/mol) is lower than C=O (715 kJ/mol) [55]. Additionally, the conjugated structure causes the simultaneous hydrogenation of the C=O and C=C groups, often resulting in a mixture of three hydrogenated products; cinnamyl alcohol (COL), hydrocinnamaldehyde (HCAL), and hydrocinamyl alcohol (HCOL) [56,57]. Furthermore, the benzene ring consists of a closed conjugated double-bond system and is usually stable [58]. CoNP catalysts have been reported to favor the adsorption of the C=O group and have shown a high selectivity in the α, β–unsaturated aldehyde hydrogenation [59,60]. More importantly, according to Tian et al., among non-noble metals such as Ni, Cu, and Co, only Co-based catalysts exhibit a higher tendency to C=O hydrogenation at the expense of somehow moderate activity [55].

## 2. Results and Discussion

### 2.1. Structural and Morphological Characterization

**Figure 1:**
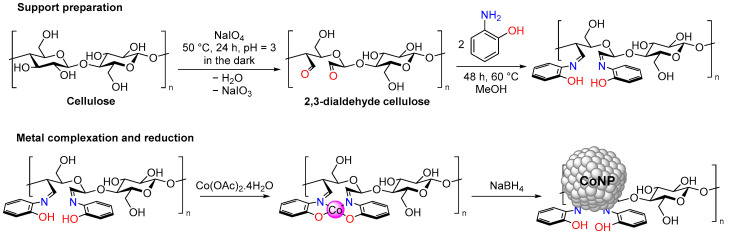
Multi-step preparation of the catalysts.

The Co@DAC-OAP catalyst was produced through a multi-step process. Initially, bleached palm pulp (BP) was oxidized using sodium periodate in an acidic environment to produce a dialdehyde cellulose (Figure 1). Next, the aldehyde groups of the dialdehyde reacted with amino groups from orthoaminophenol, resulting in the formation of imine bonds. Subsequently, cobalt ions were complexed with the salen-type ligand, and the resulting complex was reduced to cobalt nanoparticles, which are stabilized by interactions with surface salen-type groups.

**Figure 2:**
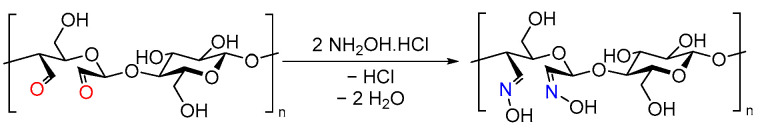
Reaction of hydroxylamine with 2,3-dialdehyde cellulose.

Regarding the first step, this methodology has previously been reported in the literature with different reaction conditions [61]. Using bleach palm pulp, optimized reaction conditions consist of a reaction time of 24 h at 50 °C with 0.5 equivalent of sodium periodate. The aldehyde group content of DAC was determined according to a literature procedure [62,63] using Schiff’s base reactions with hydroxylamine hydrochloride (Figure 2) to transform aldehyde to aldoxime. In this reaction, one equivalent of hydrochloric acid is released per aldehyde. The hydrochloric acid formed can easily be titrated by the standard pH-metric method. Using this procedure and taking into account the acidity of unoxidized bleached cellulose pulp, the content of the aldehyde group in DAC was measured to be 2.08 ± 0.1 mmol/g.

**Figure 3:**
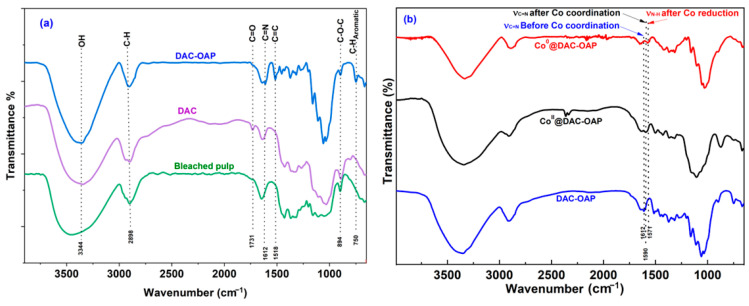
(**a**) FT-IR spectrum of the bleached pulp, DAC, and DAC-OAP; (**b**) Co@DAC-OAP.

The same Schiff base reaction, but using orthoaninophenol, leads to the formation of the bis iminophenol named DAC-OAP. To confirm the successful preparation of DAC and DAC-OAP, Fourier transform infrared (FT-IR) spectra were recorded, as shown in (Figure 3). The FT-IR analysis was used to investigate the changes in the chemical structure of cellulose at each step of the preparation process. The appearance of a distinct stretching band at 1731 cm^−1^ in the FT-IR spectrum of DAC compared to that of natural cellulose (bleached pulp), is attributed to the absorption peak of aldehyde groups [64], which confirms the successful formation of dialdehyde cellulose. Despite the successful oxidation of the cellulose pulp by sodium periodate, the characteristic absorption peaks associated with cellulose remained unchanged. Specifically, the strong broadband band observed at 3350 cm^−1^ was attributed to the vibration of -OH groups, while the intense absorption peak at 1635 cm^−1^ was assigned to the stretching vibration of absorbed water. (Figure 3a). In the FT-IR spectra of DAC-OAP, a new characteristic band at 1618 cm^−1^ belonging to C=N stretching vibration of the imine formed by the Schiff base reaction between DAC and OAP and the concomitant disappearance of the C=O band at 1731 cm^−1^ is observed [36,41]. Noteworthy, 48 h of reaction time was necessary for a total disappearance of the CO signal. Moreover, the unsaturated bond stretches of the aromatic ring at 1600~1450 (C=C) cm^−1^ and the C-H deformation band corresponding to the 1,2-substituted aromatic ring around 747 cm^−1^ are in agreement with the formation of the expected DAC-OAP.

**Figure 4:**
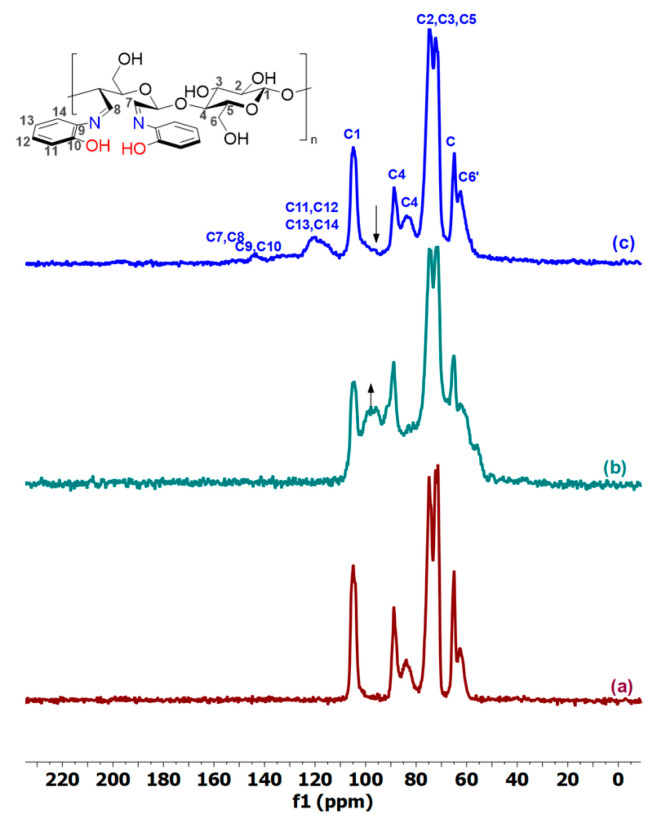
^13^C CP-MAS NMR spectra of (**a**) Cell-BP; (**b**) DAC; (**c**) DAC-OAP.

Solid-state NMR spectroscopy, and particularly ^13^C CP MAS NMR, proves to be an efficient tool for investigating the structure of chemically modified cellulose [65]. ^13^C CP-MAS NMR spectra were thus obtained for native and modified cellulose. Figure 4a–c show the spectra for natural cellulose, DAC, and DAC-OAP, respectively. In natural cellulose, signals from C1-C6 carbons span from 60 to 110 ppm. According to the literature [66], the chemical shifts observed at 62 ppm and 64 ppm correspond to the C6 and C6′ atoms of the ordered cellulose structures, and the amorphous cellulose structure, respectively, whereas the peaks observed at 71 ppm, 72 ppm, and 74 ppm represent the C2, C3, and C5 carbons of cellulose. Additionally, the C4 and C1 carbons display chemical shifts within the range of 83-88 (C4 and C4′) ppm and 104 ppm, respectively. However, the chemically modified cellulose with periodate oxidation exhibited decreases in the resonance at 104 ppm attributed to the C1 of native cellulose. According to previous works [64,67,68,69], the absence of signals in the 160–200 ppm range indicates that aldehyde groups are not present in their free form. Instead, they undergo nucleophilic addition with nearby hydroxyl groups to form hemiacetal and/or hemialdal entities, resulting in a broad signal in the 90–100 ppm region, which disappeared upon the reaction of the hidden dialdehyde cellulose with OAP. Signals from aromatic carbons were also observed between 112 ppm and 126 ppm in addition to nitrogen and oxygen ipso carbon (C9, C10) between 140 and 147 ppm. The imine C=N C7 and C8 peaks are observed as a broad signal around 155 ppm.

Formation of the Co(II) complexes with the Schiff base posed a challenge when examining the IR spectrum [70], as only minimal changes in the mid-IR range (Figure 3b) were observed. However, a noticeable shift of approximately 22 cm^−1^ in the C=N stretching band from 1612 to 1590 cm^−1^ was clearly observed upon the coordination of the cobalt ion. This was also accompanied by an intense color change (Figure S3).

Next, the FT-IR analysis was realized after the reduction of the Co(II) complexes. New signals are seen at 1571 cm^−1^ (see Figure 3b), which are attributed to the secondary amine δ -NH deformation band [71]. This confirms the expected reduction of the imine function and confirms the hydrolysis of the N-B bond during the cobalt reduction.

**Figure 5:**
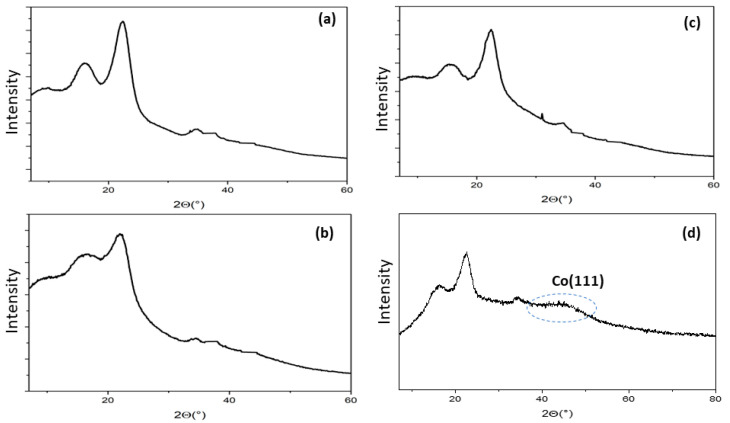
X-ray pattern of BP (**a**), DAC (**b**), DAC-OAP (**c**), and Co@DAC-OAP (**d**).

The crystallinity and the phase composition of cellulose BP, DAC, DAC-OAP, and Co@DAC-OAP were characterized using X-ray diffraction (XRD). The XRD patterns of cellulose BP (Figure 5a) show the two crystallization peaks at 2θ = 15.5° and 22.4° corresponding to the typical diffraction of the planes of (1 1 0) and (2 0 0), respectively [72]. The reduced crystallinity is a prominent characteristic of cellulose periodate oxidation. The decrease in the intensity of the crystalline peaks of the cellulose, which gives a crystallinity index of 38% for DAC versus 68% for cellulose BP (Figure 5b) is indicative of a cellulose modification. The loss of crystallinity apparently results from the opening of the glucopyranose ring and the cleavage of some glycosidic bonds, which can lead to the destruction of the ordered structure of the cellulose molecules. The crystallization peak of DAC-OAP (Figure 5c) is similar to that of DAC, indicating that the crystal structure of DAC was unchanged during functionalization. The XRD diffraction patterns obtained for Co@DAC-OAP (Figure 5d) indicated the presence of broad peaks at 40–55° diagnostic of the formation of metallic cobalt particles with a small size and a high degree of dispersion on the support material rendering CoNPs difficult to detect by XRD owing to the very small scattering angles produced by the nanoparticles that is difficult to distinguish from the background noise [73,74,75,76].

**Figure 6:**
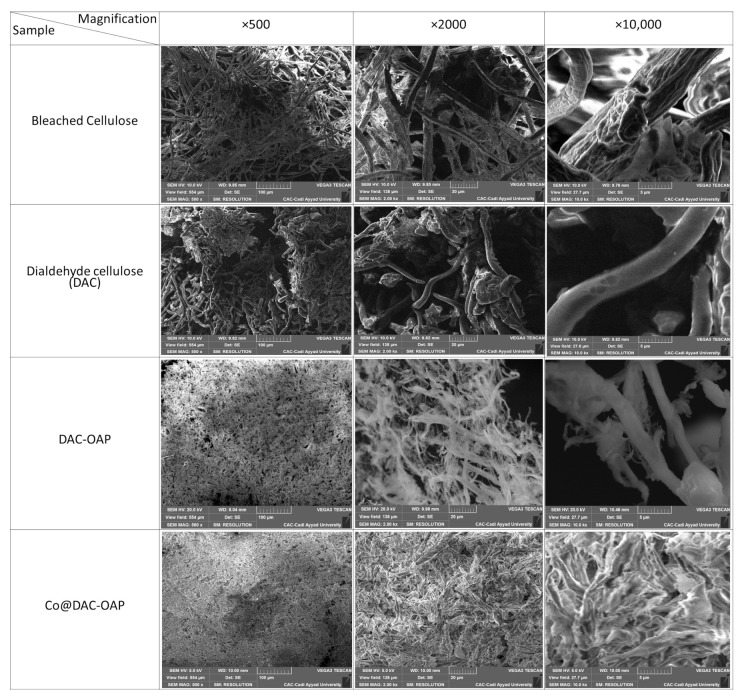
SEM images of bleached cellulose, DAC, DAC-OAP, and Co@DAC-OAP with different magnifications (×500, 2000, et 10,000).

From the SEM images of cellulose BP (Figure 6) taken as a reference with different magnifications (×500, 2000, and 10,000) the morphology of the bleached pulp consists of a rough fibrous structure with a fiber size of 7.5 µm. After the periodate oxidation, the fiber surfaces of DAC were smoother with a decrease in the diameter of the fibers up to 4 µm. After the functionalization of DAC by orthoaminophenol, the surface of DAC-OAP became rough again. In, the SEM images of Co@DAC-OAP aerogel indicate that cobalt nanoparticles are evenly distributed on the modified dialdehyde cellulose matrix and do not exhibit micrometric dimensions.

**Figure 7:**
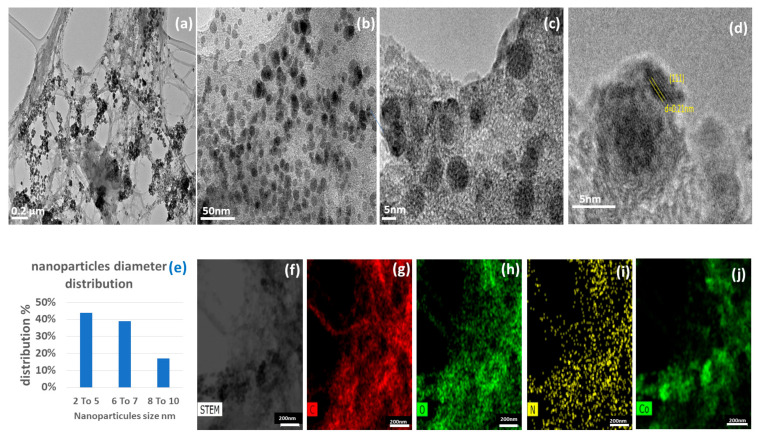
(**a**,**b**) TEM images, (**c**,**d**) HRTEM image of Co@DAC-OAP composite aerogels, (**e**) cobalt nanoparticles diameter distribution, (**f**) magnified STEM image, and (**g**–**j**) elemental mapping images were C, O, N, and Co.

TEM images of Co@DAC-OAP show spherical cobalt nanoparticles distributed over the support at (a–b) medium resolution and (c–d) high resolution; with a small size range of 2–8 nm. The lattice fringe spaces of 0.21 nm revealed in the HRTEM image (Figure 7) are assigned to the (111) plane of Co nanoparticles [77,78]. From the mean particle size value, it is possible to evaluate a cobalt dispersion of 0.16 [79]. The scanning transmission electron microscopy (STEM) and the corresponding energy dispersive X-ray spectroscopy (EDS) elemental mapping (Figure 7f–j) are obtained at a scale of 200 nm to identify the distribution of carbon, oxygen, nitrogen, and cobalt in this catalytic support. It was observed that the cobalt nanoparticles were distributed homogeneously throughout the sample, as depicted in (Figure 7f–j).

**Figure 8:**
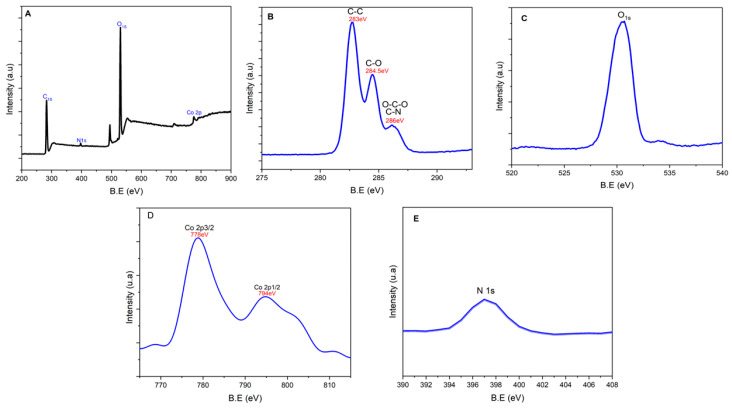
(**A**) X-ray photoelectron spectroscopy (XPS) survey analysis of Co@DAC-OAP. The fitted spectra for synthesized sample (**B**) C 1s, (**C**) O 1s, (**D**) Co 2p, and (**E**) N1s.

The surface composition and surface chemical states of Co@DAC-OAP were obtained from the X-ray photoelectron spectroscopy (XPS) data. The survey analysis of the prepared catalyst (Figure 8) confirms the presence of O, N, C, and Co. High-resolution XPS C 1s spectrum (Figure 8B) presents three peaks at 283, 284, and 286 eV. The more intense signal at the lowest binding energy corresponds to C=C Sp2 when the C 1s peak at 284 eV can be attributed to C-O. Finally, the signal at 286 eV can be assigned to the O-C-O or C-N bonds [80,81,82,83]. The oxygen in a hydroxyl group has a characteristic O1s peak in XPS Figure 8C, which is typically observed at a binding energy of around 530–532 eV depending on the specific chemical environment of the hydroxyl group, such as the presence of hydrogen bonding. Furthermore, the N1s spectrum displayed in Figure 8E reveals the presence of an absorption band at 397 eV, which can be assigned to the secondary amine (-NH) group [84,85]. According to the previous reports [24,86] the core level spectrum of Co2p Figure 8D suggests the existence of Co0 by allocating to a couple of peaks of Co 2p3/2 and Co 2p1/2 at 778 eV and 794 eV, respectively. The XPS results indicate the successful synthesis of the Co@DAC-OAP catalyst.

### 2.2. Catalytic Activity of Co@DAC-OAP

**Figure 9:**
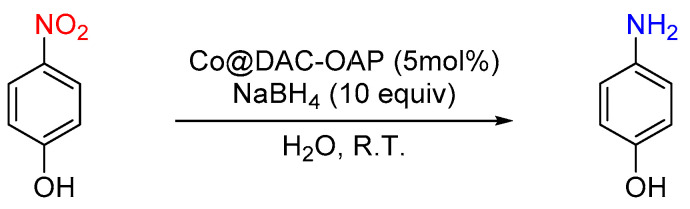
The reaction scheme for the catalytic reduction of 4-NP to 4-AP.

We then assessed Co@ DAC-OAP as a catalyst system for two reactions, the reduction of 4-nitrophenol (4-NP) to 4-aminophenol (4-AP) using sodium borohydride (NaBH_4_) as a reducing agent (Figure 9) and the hydrogenation of cinnamaldehyde using hydrogen gas (H_2_).

#### 2.2.1. Reduction of 4-Nitrophenol (4-NP)

**Figure 10:**
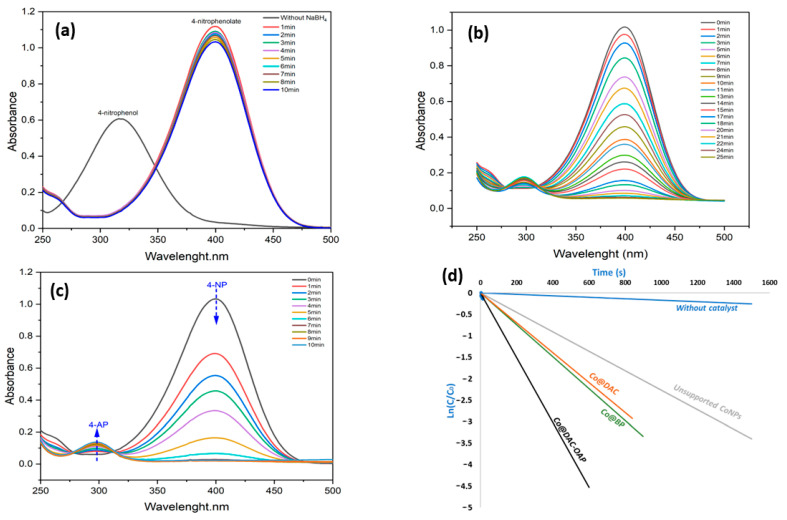
UV–vis spectra for the reduction of 4-NP with NaBH_4_ in aqueous solution: (**a**) without catalyst, (**b**) with unsupported Co (**c**) using Co@DAC-OAP and (**d**): plots of ln [C_t_/C_0_] versus reaction time for 4-NP reduction.

The progress of the reduction reaction of 4-nitrophenol (4-NP) was monitored by UV–visible spectroscopy. Figure 10 shows the results of the catalytic reduction of 4-NP to 4-AP. With 10 equivalents of NaBH_4_ and without the presence of a Co catalyst, the reduction rate was observed to be significantly low, which was evident from the minor reduction in peak intensity at 400 nm after 10 min (Figure 10a). After the addition of the 5 mol% of Co, which corresponds to 0.8 mol% of surface cobalt, to the reaction medium containing 10 equiv of NaBH_4_ (by contribution to 4-NP), a gradual disappearance of the yellow color of the medium with time is observed. Spectroscopically, this discoloration corresponds to a decrease in absorbance at 400 nm of 4-nitrophenolate with a concomitant increase in a band in the UV region at 300 nm. This new absorption band is due to 4-AP formed during the reduction. With our reaction conditions, the total conversion of 4-NP to 4-AP in the presence of Co@DAC-OAP was achieved in 7 min (Figure 10c). We also assessed the Co@BP and Co@DAC precursors, which lead to 14 and 15 min, respectively. Therefore, the modification of dialdehyde cellulose with a complexing agent is a critical step. By enhancing cellulose’s capacity to retain CoNPs, this modification enhances the catalyst stability and potentially prevents metal leaching or sintering during reactions even in water, which is not the case when particles are only stabilized with hydroxyl groups.

The catalytic data were analyzed by the following pseudo-first-order kinetic equations:

−ln(A_t_/A_0_) = −ln(C_t_/C_0_) = kt (1)

where A_0_ and At represent the absorbance of 4-NP before and after adding the catalyst for specific times, respectively, C_0_ (mol.L^−1^) is the initial concentration of 4-NP, C_t_ (mol·L^−1^) represents the concentration of 4-NP at time t (s), k (s^−1^) is the apparent rate constant.

The results of the kinetic monitoring of the catalytic systems (Figure 10d) showed a linear correlation between Ln[C_t_/C_0_] versus reaction time, indicating that the reaction is pseudo-first-order. The reaction rate constants (k) determined from the slope of the Ln (C_t_/C_0_) vs. time is 8.7 × 10^−3^ s^−1^. The catalytic performance of Co@DAC-OAP in nitrophenol reduction was found to be superior to that of other cobalt-based catalysts prepared or reported in the literature (Table S2) [49,50,78,87,88,89,90,91,92].

#### 2.2.2. Selective Hydrogenation of Cinnamaldehyde

**Figure 11:**
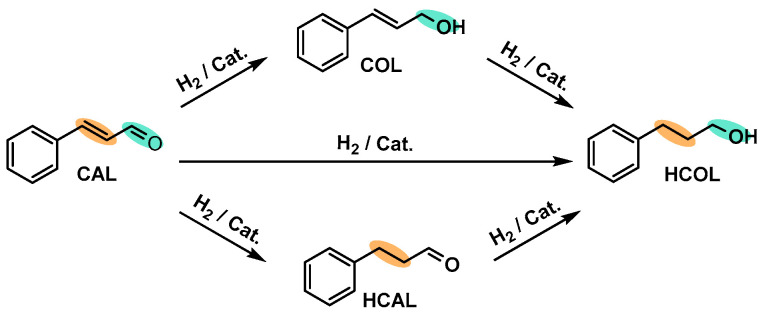
Reaction routes of CAL hydrogenation.

This reaction was selected due to its sensitivity to the structural and electronic properties, particularly the C=O group (Figure 11). While the hydrogenation of the C=C group is thermodynamically favorable compared to the C=O group, the C=O group can be adsorbed onto catalysts such as Pt, Ir, Co, and similar metals, and subsequently hydrogenated to cinnamyl alcohol (COL). The presence of metallic Co plays also a crucial role in the dissociation of adsorbed molecular H_2_ and facilitated hydrogenation [59]. The higher conversion was attributed to the smaller particle size and high surface dispersion, which resulted in greater exposure of the active sites.

The catalytic performance of Co@DAC-OAP was evaluated at various temperatures, times, and H_2_ pressures, as indicated in Table 1. A blank experiment without a catalyst showed no conversion, whereas the conversions of CAL increased when the Co@DAC-OAP catalyst was added to the mixture. The conversion was observed to increase with reaction time. Initially, at 120 °C and 5 bar of H_2_, the conversion was 36% after 3 h, and it ultimately reached 98% after 7 h. When considering only surface cobalt TOF of 75.0, 87.5, and 85.5 h^−1^ are obtained at 3, 5, and 7 h, respectively. This result suggests an activation process at the beginning of the reaction. The somehow lower TOF at a higher reaction time may be explained by deactivation or mass transfer limitation with the presence of adsorbed products. Notably, the selectivity towards COL gradually increased from 58% at 3 h to 81.5% at 7 h. Conversely, the selectivity of the other two products decreased over the course of the reaction. Specifically, the selectivity of HCAL declined from 30% to only 6.5%. These results strongly suggest a change in behavior during the course of the reaction. It may be due to a poisoning of the surface or on the contrary to an activation as suggested by the increase in TOF. The latter hypothesis would be consistent with the XPS results where a partial oxidation of the surface is observed when the particles are handled in air. Thus, a reduction step in the reaction conditions (3 h, 5 bar, 120 °C) was attempted before the introduction of the substrate. The results corresponding to the entry 2* clearly indicate that even at this low temperature the surface is reduced and the expected affinity for the carbonyl groups is almost total. With regards to pressure, as it increased from 5 to 10 and 20 bar, after 5 h of reaction time at 120 °C, the conversion indeed increased however, the selectivity in COL decreased, while the selectivity of HCAL and especially HCOL increased. Additionally, at 5 bar of H_2_ and after 5 h of reaction time, it was observed that both the conversion and the selectivity in COL increased along with the temperature. Again, this phenomenon is tentatively explained by an easier reduction of particles surface at higher temperatures. In the optimum reaction condition, Co@DAC-OAP exhibited the highest CAL conversion, with a selectivity up to 86% in COL, which is significantly higher than that achieved by all other Co-supported catalysts except that prepared by Liu et al. [93] using cobalt particles supported onto N-doped carbon layers (Co@CN-900), which used a soft proton source in the form of n-hexanol, and a low catalyst loading leading to a reaction time of 48 h to achieve full conversion, which corresponds roughly to a TOF of 2 h^−1^ when only considering only surface cobalt (Table S3). Comparison with other Co systems can also be found in Table S3 [57,59,93,94,95,96].

#### 2.2.3. Study of Catalyst Recyclability

**Figure 12:**
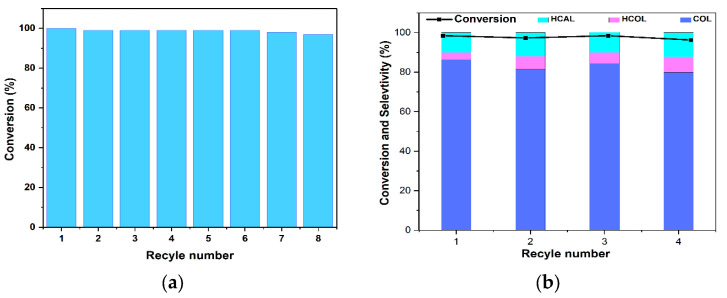
(**a**) Recyclability study of Co@DAC-OAP catalyst for nitrophenol reduction. (**b**) The reusability of Co@DAC-OAP catalyst for hydrogenation of CAL.

For the practical application of heterogeneous catalytic systems, the stability and the recyclability of the catalyst is an important factor. To evaluate the recyclability of our catalyst Co@DAC-OAP successive cycles of the catalytic reduction of 4-NP and the selective hydrogenation of cinnamaldehyde were carried out. Regarding 4-NP reduction, as shown in (Figure 12a), the recovery process can be repeated in eight successive cycles with large conversion efficiencies over 97%. The catalytic process was achieved within 10 min for the first run and 12 min for the eight runs, indicating that The Co@DAC-OAP exhibited similar catalytic performance without significant reduction, revealing that the as-prepared Co@DAC-OAP catalysts were stable. The recyclability of the Co@DAC-OAP for the cinnamaldehyde hydrogenation was evaluated at 120 °C and 5 bar for 7 h. High conversions are reached in these conditions. Thus, similarly to the recyclability study effectuated by Liu et al. or Tian et al. at high conversion, the aim is not to evaluate a loss of activity, a study at lower conversion would have been performed [58,93]. Here, we focused our interest on selectivity, which is dependent on the conversion and is meaningful only at high conversion. In order to recover the catalyst, a simple process of centrifugation and multiple ethanol washes was used. The catalyst has been recycled four times without significant loss of CAL conversion as expected while maintaining good COL selectivity. The conversion remained at around 95%. Furthermore, selectivity towards cinnamyl alcohol (COL) remained above 80% at all times, without any significant decrease, as shown in (Figure 12b). According to the mechanism suggested [93] to reach high COL selectivity, the size of the particles remains constant with a low or no sintering process occurring.

#### 2.2.4. Origin of the Selectivity Observed during the Hydrogenation of Cinnamaldehyde Catalyzed by Co@DAC-OAP

**Figure 13:**
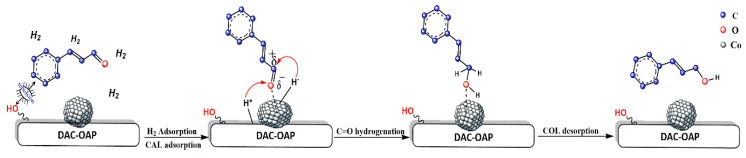
Proposed mechanism for the transfer hydrogenation of cinnamaldehyde to COL catalyzed by Co@DAC-OAP catalyst.

According to the literature [97], the hydrogenation of cinnamaldehyde using cobalt particles as a catalyst involves the reduction of the aldehyde functional group (-CHO) to the corresponding alcohol (-CH_2_OH) using molecular hydrogen (H_2_). Cobalt supported on modified dialdehyde cellulose acts as a catalyst to facilitate this reaction. A suggested mechanism is presented in (Figure 13). Indeed, the hydrogenation reaction starts with the adsorption of cinnamaldehydes molecules onto the surface of the cobalt particles through weak interactions with the metal surface. According to the results reported by [98], a significant number of oxygen-containing groups on the catalyst’s surface repelled the phenyl ring, causing the C=O bond to approach the metallic catalyst instead of the C=C bond. The small cobalt particle size observed with this synthetic procedure is determinant to maximize the phenyl repulsion and obtain an enhanced selectivity for COL with this catalytic process. The presence of basic nitrogen species in the vicinity of Co nanoparticles may facilitate the heterolytic cleavage of molecular H_2_, dissociating into hydride H^−^ and proton H^+^. Consequently, the proton will be located at the basic nitrogen site and the hydride will be located at the Co nanoparticle site [97,99,100]. Next, the nucleophilic hydride migrates from the cobalt surface to the electrophilic carbonyl carbon of the adsorbed cinnamaldehyde molecule, resulting in the formation of an adsorbed alkoxide intermediate, which undergoes further hydrogenation by accepting additional electrophilic H^+^ ion moves from the surface to the nucleophilic carbonyl oxygen leads to the formation of the desired product. The alcohol product desorbs from the cobalt surface, allowing for new reactant molecules to adsorb and continue the catalytic cycle.

## 3. Materials and Methods

### 3.1. Reagents

Cellulose was extracted from the date palm waste, sodium hydroxide (NaOH), sodium metaperiodate (NaIO_4_), hydroxylamine hydrochloride, 4-nitrophenol, ethylene glycol, 2-aminophenol (OAP), and cinnamaldehyde (CAL) and other solvents were obtained from Sigma Aldrich (St Quentin Fallavie, France) and used as received. Cobalt (II) acetate tetrahydrate was supplied by Fluka (St Quentin Fallavie, France). Acetic acid and sodium borohydride were purchased from VWR CHIMICALS (Rosny-sous-Bois, France). 

### 3.2. Catalyst Preparation

#### 3.2.1. Preparation of Cellulose: Date Palm Pulp

The bleached cellulose pulp was prepared according to the literature procedure [101]. The plant material from the date palm leaflet is coarsely ground and dried. The obtained fibers are extracted for 24 h with 200 mL of acetone using a Soxhlet apparatus, in order to remove pigments, lipids, waxes, and all substances soluble in organic solvents. The decolorized fibers were sieved with a 400 µm sieve, followed by a chemical treatment with NaOH 3% (*w*/*v*) (three times at 70 °C). Then, collected fibers are bleached using a solution of NaClO_2_ at 1.7% (*w*/*v*) in acetic buffer solution (pH = 4.8) and heated to 70 °C for two hours. This treatment allows for eliminating the lignin due to the oxidizing power of sodium chlorite. At the end, the suspension is filtered and then washed abundantly with water. The washing operation is repeated several times until the color of the cellulose fibers becomes white.

#### 3.2.2. Preparation of DAC (2,3-Dialdehyde Cellulose)

The DAC was obtained by oxidative reaction of sodium metaperiodate on bleached cellulose (Figure 2). The preparation of DAC was performed according to a previously published method with minor modifications [62]. Briefly, pulp of palm date, 2 g was dispersed in 100 mL of distilled water and then 1.3 g (6.077 mmol) of light-sensitive sodium metaperiodate (0.5 equiv. per anhydroglucose units) was added. The pH of mixture was adjusted to 3.0 with hydrochloric acid solution (0.1 M). To prevent light degradation, the mixture containing periodate was meticulously covered with aluminum foil. The reaction mixture was gently stirred 500 rpm at 50 °C in the dark for 24 h. To obtain DAC, the reaction was halted by adding 1 equivalent of ethylene glycol and subjected to multiple washes through centrifugation, washing, and redispersion in water. The removal of soluble aldehyde by-products of the resulting DAC was confirmed through negative Tollens Reagent Test of the wash water, as mentioned in the supporting information (Figures S1 and S2) [102,103,104,105]. The purified DAC samples were stored in water at 5 °C as a stable suspension for further use.

#### 3.2.3. Preparation of DAC-OAP

The cellulosic (salen) ligand (DAC-OAP) was prepared by a Schiff base reaction between the aldehyde groups of DAC and the amine groups of orthoaminophenol (OAP). A 0.6 g amount of DAC (1.24 mmol of aldehyde), 2 equiv OAP (2.48 mmol), and 30 mL of absolute ethanol were mixed in a 100 mL flask and the pH was adjusted to 3 with acetic acid solution. The mixture was allowed to react under stirring at 60 °C for 48 h. When the reaction was completed, the resulting yellow powder was separated from the solution by centrifugation and washed with methanol to remove the remaining OAP. The control of this washing was performed by UV–visible spectroscopy. The OAP is considered totally eliminated from the matrix when no characteristic OAP band in the 200 to 600 nm range is detected in the washing solution (Figure S4). After freeze-drying for 24 h, the product was obtained as a yellow powder.

#### 3.2.4. Synthesis of Cellulosic Co(salen) Complex (Co@DAC-OAP)

Cobalt(II) acetate tetrahydrate (51 mg, 0.208 mmol) is dissolved in 10 mL of distilled water solution and mixed with 0.2 g of DAC-OAP. The mixture was sonicated for 15 min and then left under magnetic stirring for 20 h. The resulting product undergoes freeze-drying for 24 h to obtain aerogels. Then salen complexed Co(II) in the DAC-OAP matrix were reduced to cobalt (0) by NaBH_4_. The aerogel color immediately changed from pink to black. Several washings were performed and then, the water was removed by lyophilization after freezing the hydrogel aerogels at −20 °C for one night. Cobalt loading (5.7 wt%) was then assessed by ICP analysis (realized at UMET–UMR 8207 laboratory).

#### 3.2.5. Synthesis of Cellulosic Co@BP and Co@DAC

The identical procedures that were previously described were used to prepare a cobalt catalyst supported on unoxidized BP and unmodified dialdehyde cellulose.

### 3.3. Catalyst Characterization

The aldehyde group content of DAC. Schiff’s base reactions with hydroxylamine hydrochloride to transform aldehyde to aldoxime release one equivalent of HCl that was quantified. Briefly, a suspension of never-dried dialdehyde cellulose of known mass percentage was mixed and suspended with 25 mL of hydroxylamine hydrochloride (0.2 M) and stirred at 40 °C for 18 h. The hydrochloric acid formed was titrated by the pH-metric method. A blank measurement was performed in a similar manner with unoxidized bleached cellulose pulp.

The aldehyde content is determined from the following equations:

(2) $$\mathrm{AC}\;(\mathrm{mmol}/g)=\frac{\left(C_{NaOH}\times (V_{DAC}-V_{BP})\right)}{m}$$

where m = mass of sample, C_NaOH_ = concentration of the NaOH solution, V_DAC_ = equivalence volume with oxidized bleached pulp, and V_BP_ = equivalence volume with non-oxidized bleached pulp.

FT-IR spectroscopy. Spectra of dried powders were obtained at room temperature on a Perkin Elmer Spectrum 1000 FT-IR (Casablanca, Morocco) spectrometer under continuous dry air flow. For sample analysis, a KBr pellet was prepared to serve as a background spectrum, and pellets containing our sample to be analyzed were prepared in a KBr matrix (Sigma Aldrich, St Quentin Fallavie, France) (4 mg of ground product in 396 mg of KBr). FT-IR spectra were recorded with 32 scans between 450 and 4000 cm^−1^ at a resolution of 4 cm^−1^.

X-ray diffraction. The XRD analysis was performed with a Rigaku model, and SmartLab X-ray diffractometer to identify the crystal structure of the samples with the scanning angle ranging from 5° to 60°.

The crystallinity was calculated using the empirical Segal equation [106]

(3) $$I.C=\frac{I_{\left(002\right)}-\;I_{\mathrm{am}}}{I_{002}}$$

With I_002_ the maximum diffraction intensity of the d_002_ plane at 2θ = 22° and I_am_ the diffraction intensity for the amorphous portion at 2θ = 18°.

Scanning electron microscope. SEM was used to examine the surface morphologies of bleached pulp (Cell-BP), DAC, modified dialdehyde cellulose (DAC-OAP), and the catalyst. The (Vega 3 TESCAN) with conventional tungsten was used to perform SEM analysis.

UV–visible spectroscopy. UV–vis spectrometer (Perkin Elmer Lambda 650) was used for the kinetic study.

Gas chromatography. Reaction products were identified by GC (QP2010 Plus SHIMADZU, Marne la Vallée, France) equipped with an SPB-MS column (30 m length, 0.25 mm diameter). Reactant and reaction products were quantified by GC (SHIMADZU GC-2014) equipped with a flame ionization detector and a ZB-5 column (30 m length, 0.25 mm diameter).

Solid-state NMR spectroscopy. ^1^H MAS NMR and ^13^C CP-MAS NMR experiments were measured using a BRUKER Avance 400 spectrometer operating at 400 Mhz and 100.6 MHz respectively (Bruker France, Palaiseau France), using the combination of cross-polarization, high-power proton decoupling and magic angle spinning (CP/MAS) methods. The spinning speed was set at 12.5 kHz.

Transmission electron microscopy. Transmission electron microscopy (TEM) was used to determine the size and shape of the synthesized cobalt nanoparticles. The analysis was performed using an FEI Tecnai G2 (Eindhoven, Netherland), operated at an accelerating voltage of 200 kV was used for the morphological observation. TEM sample was prepared by suspending the catalytic system in distilled water. Then, three drops were placed on carbon-coated electron microscope grids, then air-dried and observed in TEM.

X-ray photoelectron spectroscopy. XPS analyses were carried out by a Kratos AxisUltra DLD spectrometer equipped with an Al-Kα mono-chromatized X-ray source (1486.6 eV) operating at 180 W. Survey spectra were obtained at a pass energy 160 eV and C 1s, O 1s, and Co 2p core-level spectra at a pass energy 20 eV, with a 100 meV energy step and 150 ms dwell time. Spectra were analyzed with CasaXPS software (Version 2.3.25PR1.0).

### 3.4. Catalytic Test

Methodology for catalytic reduction of 4-nitrophenol. The catalytic reduction of 4-nitrophenol to 4-aminophenol was performed at room temperature with the presence of Co@DAC-OAP as catalyst. A 10 mL amount of 4-nitrophenol (0.5 g/L) solution was mixed with 10 equiv of NaBH_4_. Then 5 mol % of catalyst was added to the solution and a disappearance of the yellow color of nitrophenolate with time was observed. UV–vis absorption spectra were recorded until complete disappearance of the nitro group peak at 400 nm and the 4-nitrophenol concentration was determined at each time point according to the calibration curve.

The catalytic materials can be easily separated from the substrate/product solution by centrifugation at 15,000 rpm for 5 min.

Selective catalytic hydrogenation of cinnamaldehyde. The catalytic hydrogenation of cinnamaldehyde was performed at 80–140 °C and the hydrogen pressure was adjusted to the required pressure in an autoclave with a capacity of 50 mL, which was loaded with 5 mL ethanol, 0.3 mL CAL, and 30 mg of catalyst (1%) that had been thoroughly washed with water to eliminate the residual NaBH_4_ and dried under vacuum. The reaction was performed for 3–7 h while stirring the reaction mixture at 200 rpm. After each reaction, the autoclave was cooled to room temperature. The catalyst and reaction products were separated by centrifugation. The products of the reaction were identified by GC–MS, and the amount of reactants and products were monitored by gas chromatograph.

## 4. Conclusions

In conclusion, we have developed a new catalyst support based on cellulose extracted from date palm waste. Using this support, we have prepared a heterogenous cobalt catalyst. The catalyst has been characterized for its phase composition and surface morphology using various methods. The Co@DAC-OAP exhibited excellent catalytic activity for the 4-nitrophenol reduction using NaBH_4_ as a reducing agent and for the cinnamaldehyde selective hydrogenation using H_2_. The results showed that the Co@DAC-OAP system which had the catalytic activity (TOF = 1060 h^−1^) and the pseudo-first-order rate constant (k = 8.7 × 10^−3^ s^−1^) had the largest catalytic performance for the reduction of 4-nitrophenol, compared with those of the Co-NPs and the composites Co@DACs and Co@PB. Furthermore, the catalyst was found to be stable in water with excellent catalytic ability even after eight successive recycles with more than 97% reduction efficiency for nitrophenol reduction. The catalyst also performed excellent activity, selectivity, and stability, with a 97% conversion of CAL hydrogenation (TOF = 87 h^−1^) and 86% COL selectivity. Notably, no significant deactivation was observed even after four cycles in the reducing atmosphere of H_2_, which is known to favor the sintering of particles. Thus, the prepared Co@DAC-OAP catalyst shows remarkable catalytic performances, surpassing other tested catalysts in terms of catalytic performance, stability, and recyclability. These findings highlight its potential as a sustainable and efficient catalyst for various important chemical transformations.

## Figures and Tables

**Table 1:** Influence of different reaction parameters on the activity and the selectivity of supported cobalt catalyst in the hydrogenation of cinnamaldehyde.

Entry	Co:CAL	Substrate	Tem. (°C)	P(H_2_) (bar)	Time (h)	Conv. (%)	Sel. (%)		
							COL	HCAL	HCOL
1	0:100	CAL	120	5	7 h	4.5	48.9	51.1	0
2	1:100	CAL	120	3	7 h	50.3	74	9.16	16.5
2 *	1:100	CAL	120	3	7 h	49	90.6	Traces	9.3
3	1:100	CAL	120	5	7 h	96	81.5	6.5	12
4	1:100	CAL	120	5	5 h	70	71	14	15
5	1:100	CAL	120	5	3 h	36	58	30	12
6	1:100	CAL	120	10	5 h	80.8	65.6	24.8	9.6
7	1:100	CAL	120	20	5 h	99	44.3	12.4	43.3
8	1:100	CAL	140	10	3 h	48.8	27.6	54	18.4
9	1:100	CAL	140	10	5 h	94.9	53.6	10.4	36
10	1:100	CAL	140	10	7 h	100	24	0	76
11	1:100	CAL	60	5	5 h	<5	0	100	0
12	1:100	CAL	80	5	5 h	17%	12	83	5
13	1:100	CAL	100	5	5 h	48	44	16	40
14	1:100	CAL	140	5	5 h	89	78.6	11	10.4

* Addition of the catalyst activation step under 10 bar H_2_ for 3 h at 120 °C.

## Data Availability

Data are contained within the article and Supplementary Materials.

## Supplementary Materials

The following supporting information can be downloaded at: https://www.mdpi.com/article/10.3390/molecules29081734/s1.
