# Supplementary material for: Schiff Base Functionalized Cellulose: Towards Strong Support-Cobalt Nanoparticles Interactions for High Catalytic Performances
Source: Molecules. 2024 Apr 11;29(8):1734. doi: 10.3390/molecules29081734 (PMC11051967; doi:10.3390/molecules29081734)
Supplement: Supplementary file 1 [file molecules-29-01734-s001.zip › molecules-2916342-supplementary.pdf]

molecules

Schiff Base Functionalized Cellulose: Towards Strong Support-Cobalt Nanoparticles Interactions for high catalytic performances

* Correspondence: h.kaddami@uca.ma (H.K); nicolas.merle@univ-lille.fr (N.M)

Supporting Information

Contents:

1. Control of byproduct during periodate oxidation of cellulose 1
2. reaction scheme of the Co@DAC-OAP catalyst preparation 2
3. UV-Vis monitoring of the DAC-OAP washing after the reaction 3
4. FTIR ANALYSIS: Cobalt supported on unmodified DAC (Co@DAC).................................3
5. FTIR ANALYSIS: Cobalt supported on modified DAC (Co@DAC-OAP) 4
6. Performance comparisons of our catalyst with catalysts reported in the literature 6
7. References...6

1. Control of byproduct during periodate oxidation of cellulose

Periodate oxidation of cellulose can also lead to depolymerization of polysaccharides, which depends on the concentration of $NaIO_4$, temperature, time, and pH. The literature describes the formation of several by-products during the oxidation of cellulose [1,2]. **Error! Reference source not found.** shows the structures of the by-products present in the mixture during the reaction.

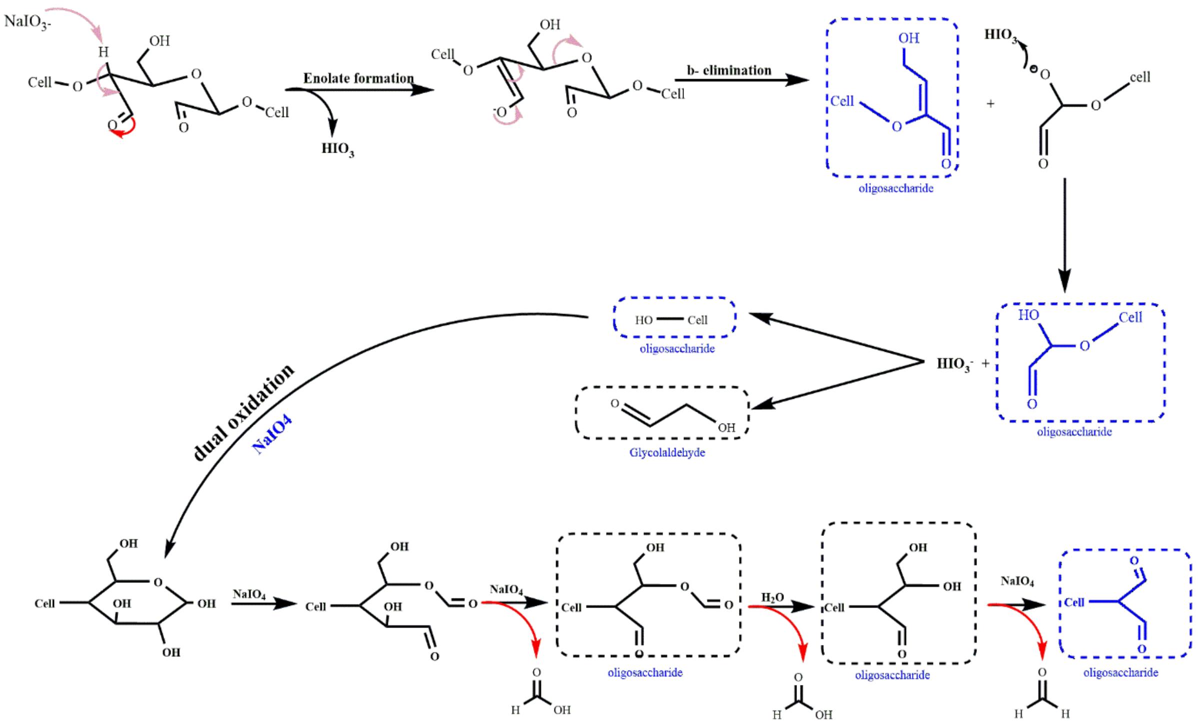

Figure S1. Structures of depolymerization products (oligosaccharides) possible during the periodate oxidation of cellulose**.**

To ensure that undesirable by-products were significantly removed from the reaction mixture, a control by Tollens' reagent was performed (3) . This reagent was used to check for the presence of soluble aldehyde functions in the supernatants during centrifugal washing. For this purpose, 5 drops of Tollens' reagent were added to 2 ml of supernatant liquid from each wash in a test tube and then the tube is placed in a water bath. During the reaction; the silver ion oxidizes the aldehyde to give a carboxylate ion according to the following redox reaction:

$$R\text{-}CHO + 2Ag^{+}(aq) + 3HO^{-} \rightarrow RCOO^{-} + 2Ag(s) + 2H_2O$$

In this process, R–CHO can react with Tollen's reagent to form a silver mirror (**Error! Reference source not found.**). The positive result indicates the presence of aldehyde (4).

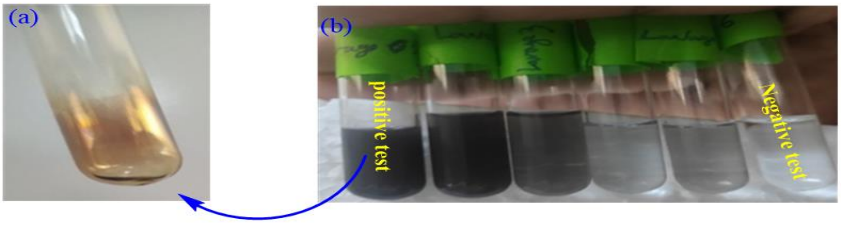

Figure S2. (a)- silver mirror on the test tube of the positive tollens test (b)- tests of the tollens of each wash.

2. Reaction scheme of the Co@DAC-OAP catalyst preparation

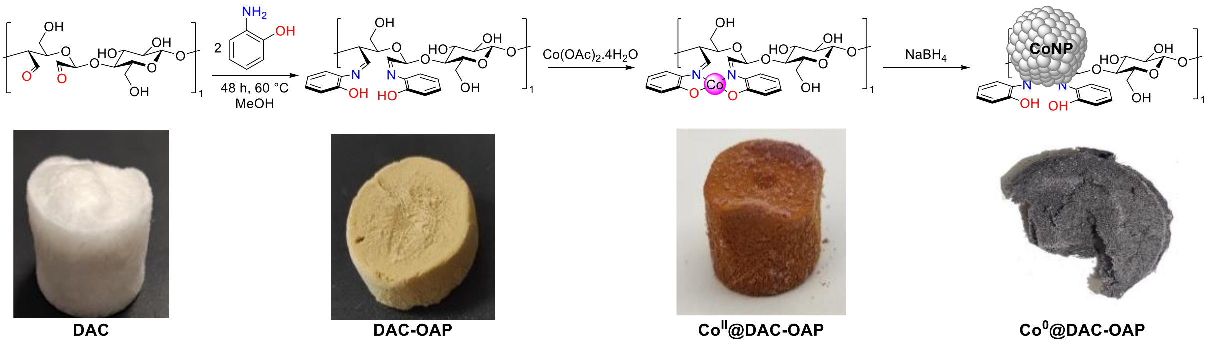

Figure S3. reaction scheme of the Co@DAC-OAP catalyst preparation. .

3. UV-Vis monitoring of the DAC-OAP washing after the reaction

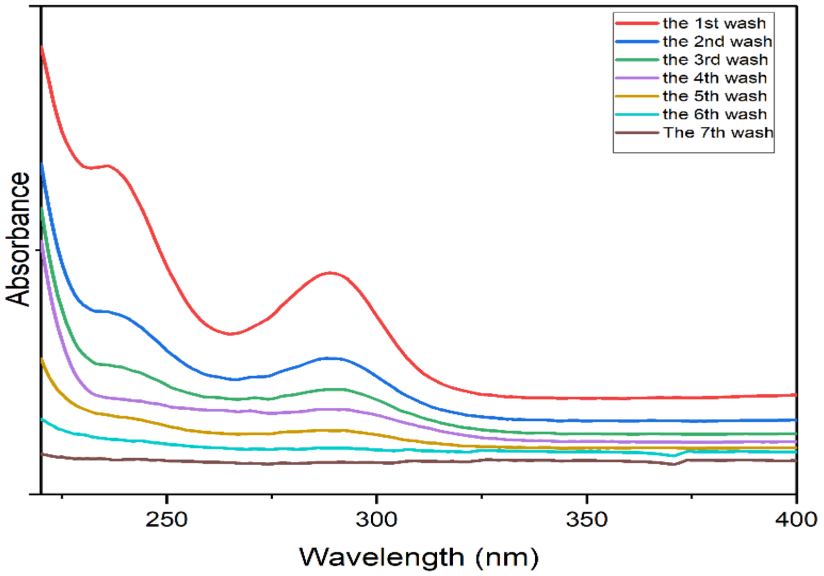

Figure S4. The UV-vis spectra obtained from the sequential washes of the DAC-OAP after the reaction occurred in the supernatant.

4. FTIR ANALYSIS: Cobalt supported on unmodified DAC (Co@DAC)

The stability and cycling performance of the catalyst prepared by using the aldehydes group of dialdehyde cellulose to immobilize cobalt nanoparticules (Co@DAC) can be studied under the same conditions for reducing nitrophenol to aminophenol. It can be observed from the Fig. 4 that the Co@salen (Co@DAC-OAP) has no significant decrease in performance after eight cycles of use. In comparison, after being recycled once, the catalytic performance of Co@DAC drops dramatically from 90% to 78 % over an 8 min reaction period (**Error! Reference source not found.**), which may attribute to the leaching of metal from DAC surface and the fact that the aldehyde can be converted to the corresponding primary alcohol during the reduction process of the cobalt ions under standard conditions (**Error! Reference source not found.**).

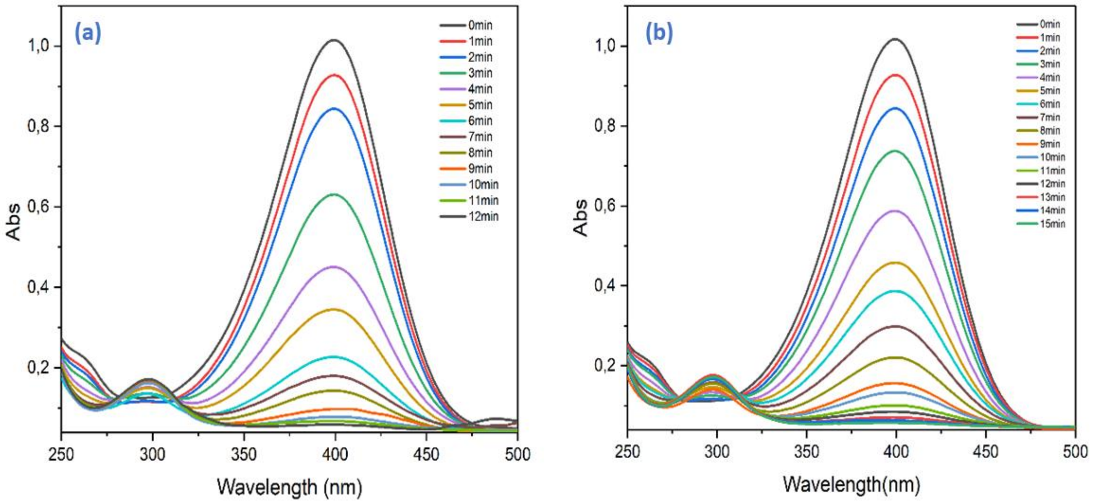

Figure S5. UV–Vis spectra for the reduction of 4-NP with $NaBH_4$ using Co@DAC: (a) 1st use of catalyst, (b) 2nd use of catalyst**.**

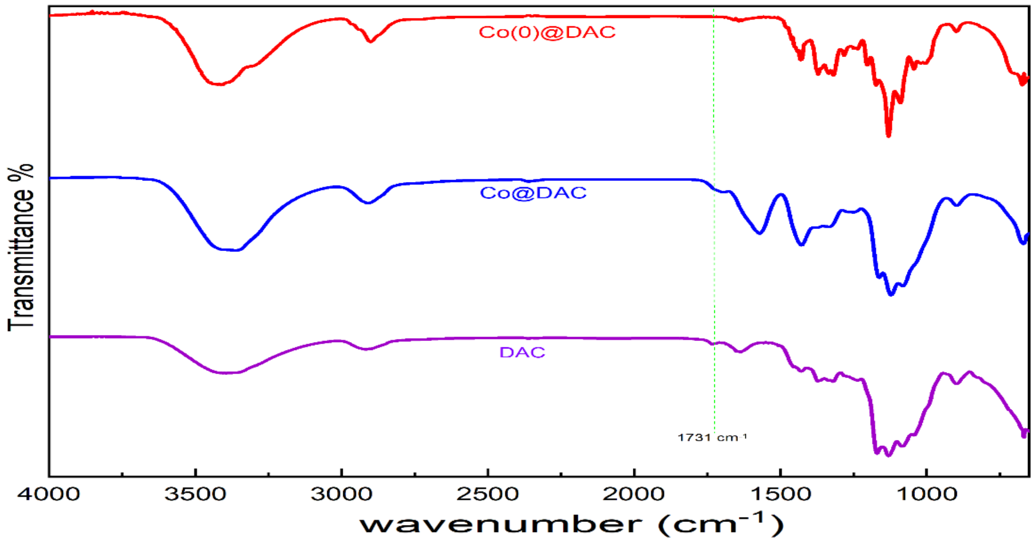

Figure S6. IR Spectrum of dialdehyde cellulose, its cobalt complexation and reduction.

5. FTIR ANALYSIS: Cobalt supported on modified DAC (Co@DAC-OAP)

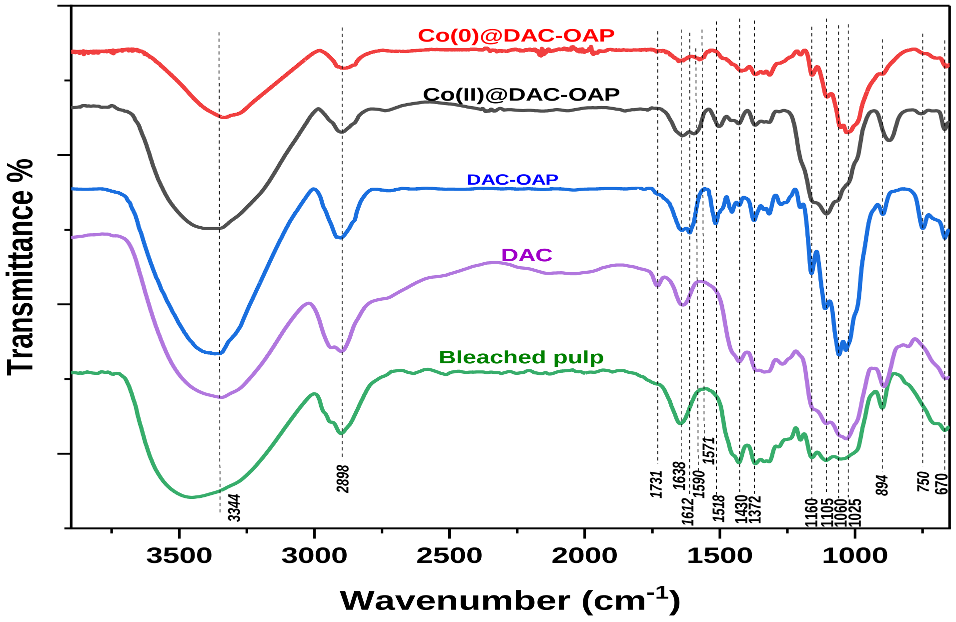

Figure S7. IR spectrums of cellulose and its derivative during the oxidation, modification, complexation.

Table S1. FTIR band assignments of cellulose and its derivative during the oxidation, modification, complexation**.**

Wavenumber (cm^{-1})	Band assignment*
**3340**	O-H stretching vibration
**2898**	stretching vibration of C-H
**1731**	C=O aldehyde
**1638**	absorbed water (hydrogen-bonded)
**1612**	C=N stretch
**1590**	C=N after complexation with cobalt
**1571**	bending vibrations of the-NH- after reduction of C=N
**1518**	C=C stretches in the aromatic ring
**1430**	deformation or asymmetric C-O stretch of C-O stretching
**1372**	deformation vibrations of C-H
**1160**	C-O-C at glycosidic linkage
**1106**	ring in plane stretching
**1060**	C-O of secondary alcohols
**894**	COC at glycosidic linkage; amorphous region
**750**	$C-H_{Aromatic}$ bending
**670**	C-OH out-of-plane bending mode

*(5) (6) (7)(8).

6. Performance comparisons of our catalyst with catalysts reported in the literature

Table S2. Comparison of the reducing ability of Co@DAC-OAP in reduction of nitrophenol with other reported Co systems.

Catalyst systems	Concentration (M)		% molar or Catalyst amount	Co NPs size (nm)	K (min^{-1})	cycle number	Reference
	4-NP (M)	$NaBH_4$					
**TBAB stabilized Co NPs**	2×10^{-5}	2×10^{-5}	3mg	90–95	0.21 (29°C)	5	(9)
**Co@N-Doped carbon**	$0{,}2\times10^{-3}$	0,0263	23.8%	8	0,96	--	(10)
**LDO-supported cobalt**	$0{,}2\times10^{-3}$	16×10^{-3}	15%	6.8-11.1	1	8	(11)
**Co/RGO**	0.096×10^{-3}	0,1	1.2 g/L	8	0.03	6	(12)
**$Co_{0.85}$ Se**	0.5×10^{-3}	0.02	0.1g/l	-	0.331	5	(13)
**Co-CC**	0.16×10^{-3}	0.2	0.27 g/l	15–30	0,42	5	(14)
**p(AMPS)–Co**	1.44×10^{-2}	2.88×10^{-1}	15%	100	0.1184 (30 °C)	5	(15)
**$Co@SiO_2$**	0.6×10^{-3}	$0{,}3\times10^{-3}$	13,2%	20	0,8	4	(16)
**Cobalt Microflowers**	6.15×10^{-5}	1.96×10^{-1}	40%	3-15	0,243	3	(17)
**Unsupported Co**	3.5×10^{-3}	35×10^{-3}	5%	-	0.13	-	This work
**Co@BP**	3.5×10^{-3}	35×10^{-3}	5%	-	0,21	3	This work
**Co@DAC**	3.5×10^{-3}	35×10^{-3}	5%	-	0,22	3	This work
**Co@DAC-OAP**	3.6×10^{-3}	36×10^{-3}	5%	2-7	0.522	8	This work

Table S3. Catalytic performance of various Co catalysts in the selective hydrogenation of CAL to COL.

Catalysts	Co content (%)	T(ºC)	P (MPa)	t (h)	Conv. (%)	Sel. (%)	Reference
**Co/ZSM-5**	16.7	100	2.0	6	99.10	34.90	(18)
**Co/TiO_2**	15	120	1.0	1	27.10	58.00	(19)
**Co@CN-900**	38.7	80	n-hexanol as a proton donor	48	> 99	99.00	(20)
**$CoRe/TiO_2$**	1.98	160	Formic acid as a proton donor	12	96	82.00	(21)
**Co/SiO_2**	15	120	1.0	1	20.00	38.00	(19)
**Co/Al_2O_3**	15	120	1.0	1	10.00	17.00	(19)
**CoxSi@C-N**	31.9	90	1	4	82.5	75.1	(22)
**Co/p-BN-500**	7.98	120	0.4	4	51.98	73.1	(23)
**Co/p-BN-500**	7.98	140	0.4	2	77.40	67.80	(23)
**Co@DAC-OAP**	5,7	120	0.5	7	98.2	86.3	This work

7. References

1. Pandeirada, C. O.; Achterweust, M.; Janssen, H. G.; Westphal Y.; Schols, H. A. Periodate oxidation of plant polysaccharides provides polysaccharide-specific oligosaccharides. *Carbohydr. Polym.* **2022**, 291, 119540.
2. Pandeirada, C. O.; Achterweust, M.; Janssen, H. G.; Westphal Y.; Schols, H. A. Identification of plant polysaccharides by MALDI-TOF MS fingerprinting after periodate oxidation and thermal hydrolysis. *Carbohydr. Polym*. **2022**, 292, 119685.
3. She, Q.; Li, J.; Lu, Y.; Lin, S.; You, R. In situ synthesis of silver nanoparticles on dialdehyde cellulose as reliable SERS substrate. *Cellulose*, **2021,** 28, 10827–10840.
4. Li, J.; Wang, Y.; Zhang, Q.; Huo, D.; Hou, C.; Zhou, J.; Luo, H.; Yang, M. New application of old methods:

Development of colorimetric sensor array based on Tollen's reagent for the discrimination of aldehydes based on Tollen's reagent. *Anal. Chim. Acta*, **2020**, 1096, 138–147.

5. Yasmeen, S.; Kabiraz, M.K.; Saha, B.; Qadir, M.D.; Gafur, M.D.; Masum, S. Chromium (VI) Ions Removal from Tannery Effluent using Chitosan-Microcrystalline Cellulose Composite as Adsorbent. *Int. Res. J. Pure Appl. Chem.* **2016**, 10, 1–14.
6. Xiao, G.; Wang, Y.; Zhang, H.; Zhu, Z.; Fu, S. Dialdehyde cellulose nanocrystals act as multi-role for the formation of ultra-fine gold nanoparticles with high efficiency. *Int. J. Biol. Macromol.* **2020**, 163, 788–800.
7. El Idrissi, N.; Belachemi, L.; Merle, N.; Zinck, P.; Kaddami, H. Comprehensive preparation and catalytic activities of Co/TEMPO-cellulose nanocomposites: A promising green catalyst. *Carbohydr. Polym.* **2022,** 295, 119765.
8. Oh, S.Y.; Yoo, D.I.; Shin, Y; Seo, G. "FTIR analysis of cellulose treated with sodium hydroxide and carbon dioxide. *Carbohydr. Res.* **2005**, 340, 417–428.
9. Mondal, A.; Mondal, A.; Adhikary, B; Mukherjee, D.K. Cobalt nanoparticles as reusable catalysts for reduction of 4-nitrophenol under mild conditions. *Bull. Mater. Sci.* **2017**, 40, 321–328.
10. Butova, V.V.; Polyakov, V.A.; Erofeeva, E.A.; Rusalev, Y.V.; Gritsai, M.A.; Ozhogin, I.V.; Borodkin, G.S.; Kirsanova, D.Y.; Gadzhimagomedova, Z.M.; Guda, A.A.; Soldatov, A.V. Cobalt nanoparticles embedded in porous N-doped carbon support as a superior catalyst for the p-nitrophenol reduction. *Appl. Surf. Sci.* **2022**, 592, 153292.
11. Ma, H.; Wang, H.; Wu, T.; Na, C. Highly active layered double hydroxide-derived cobalt nano-catalysts for p-nitrophenol reduction. *Appl. Catal. B Environ.* **2016**, 180, 471–479.
12. Chen, F.; Xi, P.; Ma, C.;Shao, C.; Wang, J.; Wang, S.; Liu, G.; Zeng, Z. "In situ preparation, characterization, magnetic and catalytic studies of surfactant free RGO/FexCo100-x nanocomposites. *Dalt. Trans.* **2013**, 42, 7936–7942.
13. Zhang, Z.X.; Wang, X.W.; Wu, K.L.; Yue, Y.X.; Zhao, M.L.; Cheng, J.; Ming, J.; Yu, C.J; Wei, X.W. Co0.85Se bundle-like nanostructure catalysts for hydrogenation of 4-nitrophenol to 4-aminophenol. *New J. Chem.* **2014**, 38, 6147–6151.
14. Hasan, Z.; Cho, D.W.; Chon, C.M.; Yoon, K.; Song, H. "Reduction of p-nitrophenol by magnetic Co-carbon composites derived from metal organic frameworks. *Chem. Eng. J.* **2016**, 298, 183–190.
15. Sahiner, N.; Ozay, H.; Ozay, O.; Aktas, N. A soft hydrogel reactor for cobalt nanoparticle preparation and use in the reduction of nitrophenols. *Appl. Catal. B Environ.* **2010**, 101, 137–143.
16. Yan, N.; Zhao, Z.; Li, Y.; Wang, F.; Zhong, H.; Chen, Q. Synthesis of novel two-phase Co@SiO2 nanorattles with high catalytic activity. *Inorg. Chem.* **2014**, 53, 9073–9079.
17. Senapati, S.; Srivastava, S.K.; Singh, S.B. Synthesis, magnetic properties and catalytic activity of hierarchical cobalt microflowers. *J. Nanosci. Nanotechnol.* **2012**, 12, 3048–3058.
18. Zhang, B.; Zhang, X.B.; Xu, L.Y.; Zhang, Y.J.; Qin, Y.H.; Liang, C.F. Selective hydrogenation of cinnamaldehyde over ZSM-5 supported Co catalysts. *React. Kinet. Mech. Catal.* **2013**, 110, 207–214.
19. Joseph Antony Raj, K.; Prakash, M.G.; Elangovan, T.; Viswanathan, B. Selective hydrogenation of cinnamaldehyde over cobalt supported on alumina, silica and titania. *Catal. Letters*, **2012**, 142, 87–94.
20. Liu, X.; Cheng, S.; Long, J.; Zhang, W.; Liu, X.; Wei, D. MOFs-Derived Co@CN bi-functional catalysts for selective transfer hydrogenation of α,β-unsaturated aldehydes without use of base additives. *Mater. Chem. Front.* **2017**, 1, 2005–2012.
21. Chen, M.; Wang, Y.; Jiang, L.; Cheng, Y.; Liu, Y.; Wei, Z. Highly Efficient Selective Hydrogenation of Cinnamaldehyde to Cinnamyl Alcohol over CoRe / TiO2 Catalyst. *Molecules*. **2023**, 28, 3336.

22. Zhang, L.; Chen, X.; Li, C.; Armbrüster, M.; Peng, Z.; Liang, C. Cobalt Silicides Nanoparticles Embedded in N-Doped Carbon as Highly Efficient Catalyst in Selective Hydrogenation of Cinnamaldehyde. *ChemistrySelect,* **2018**, 3, 1658–1666.

23. Zhang, R.; Wang, L.; Yang, X.; Tao, Z.; Ren, X.; Lv, B. The role of surface NH groups on the selective hydrogenation of cinnamaldehyde over Co/BN catalysts," *Appl. Surf. Sci.* **2019**, 492, 736–745.
